# Combination of arsenic trioxide and Dasatinib: a new strategy to treat Philadelphia chromosome‐positive acute lymphoblastic leukaemia

**DOI:** 10.1111/jcmm.13436

**Published:** 2017-12-20

**Authors:** Tao Wang, Chunyan Cheng, Lijun Peng, Mengqing Gao, Mengping Xi, Sophie Rousseaux, Saadi Khochbin, Jin Wang, Jianqing Mi

**Affiliations:** ^1^ State Key Laboratory for Medical Genomics and Department of Hematology Shanghai Institute of Hematology Collaborative Innovation Center of Systems Biomedicine Pôle Sino‐Français des Sciences du Vivant et Genomique Rui Jin Hospital Shanghai Jiao Tong University School of Medicine Shanghai China; ^2^ CNRS UMR 5309, INSERM U1209 Institute for Advanced Biosciences Université Grenoble‐Alpes La Tronche France

**Keywords:** Philadelphia chromosome‐positive acute lymphoblastic leukaemia, arsenic trioxide, tyrosine kinase inhibitor, apoptosis, IRE1/JNK/PUMA pathway, ATF4

## Abstract

Tyrosine kinase inhibitors (TKIs) have significantly improved the prognosis of Philadelphia chromosome‐positive acute lymphoblastic leukaemia (Ph^+^ ALL), one of the most common and aggressive forms of haematological malignancies. However, TKI resistance has remained an unsolved issue. In this study, we investigate the impact of adding arsenic trioxide (ATO) on the action of Dasatinib, a second‐generation TKI, in Ph^+^ ALL. We show that ATO cooperates with Dasatinib in both TKI‐sensitive and resistant Ph^+^ ALL cell lines to increase apoptosis and we unravel the underlying mechanisms. Indeed, combining ATO and Dasatinib leads to severe cell apoptosis by activating the UPR apoptotic IRE1/JNK/PUMA axis, while neutralizing the UPR ATF4‐dependent anti‐apoptotic axis, activated by ATO alone. Additionally, ATO and Dasatinib in combination repress the expression of several genes, which we previously showed to be associated with shorter survival probability in ALL patients. Overall these data support the use of ATO in combination with Dasatinib as a novel therapeutic regimen for Ph^+^ ALL patients.

## Introduction

Ph^+^ ALL accounts for 20–30% of ALL. The BCR‐ABL1 fusion protein, which is encoded by the Ph chromosome, is the main driver of Ph^+^ ALL. The outcome of Ph^+^ ALL was very poor until the use of TKIs, which target the kinase domain of the ABL1 moiety and block the catalytic activity of BCR‐ABL1. However, Ph^+^ ALL patients only exhibit a transient response to a single‐agent TKI. Thus, in clinical practice, multi‐agent chemotherapy needs to be added in combination with TKIs. Nevertheless, resistance to this treatment has remained an issue and is associated with a high mortality 1. Accumulated studies point to apoptosis incompetence as a main hallmark of TKI resistance 2, 3. Therefore, new strategies should be developed to restore the apoptosis‐inducing ability of TKIs.

ATO belongs to the arsenic compound family that has been proved to be a potent molecule in fighting various cancers, including several haematological malignancies 4, 5. ATO combined with all‐trans retinoic acid (ATRA) increases the 50‐month overall survival rate of acute promyelocytic leukaemia (APL) patients to 99%, a result which is superior to ATRA combined with chemotherapy 6, 7. In addition, ATO combined with TKI showed synergistic anti‐leukaemia effect on chronic myelogenous leukaemia (CML), which is also caused by BCR‐ABL1 8, 9. Recently, we found that ATO was able to rewire the response of mantle cell lymphoma (MCL) to the proteasome inhibitor bortezomib 10. Notably, in this context, the apoptosis‐promoting effects of ATO were identified as the most prominent effects within the anti‐tumour activities of this molecule. However, whether the combination of ATO with Dasatinib also induces synergistic effects to trigger apoptosis in Ph^+^ ALL has not yet been studied.

Apoptosis is a highly regulated process involving pro‐ and anti‐apoptosis factors that form complex networks driving cell fate. Proteins of the Bcl‐2, IAP and Flip families are critical regulators of apoptosis. As a member of the Bcl‐2 family, p53‐up‐regulated modulator of apoptosis (PUMA) induces mitochondrial dysfunction and caspase‐dependent apoptosis by binding to anti‐apoptosis Bcl‐2 family members and by relieving the pro‐apoptosis proteins Bak/Bax 11. PUMA is activated by a wide variety of stress signals, such as Endoplasmic Reticulum (ER) stress, and acts as an important mediator between ER stress and apoptosis 12.

Unfolded protein response (UPR), which is mediated by three axes, respectively PERK/eIF2α/ATF4, ATF6 and IRE1/XBP1s, is a conserved protective process that counteracts ER stress. However, under the condition of unrecoverable ER stress, UPR is also able to trigger apoptosis.

We made the hypothesis that an interesting therapeutic strategy would be to induce UPR pro‐apoptotic pathways, while inhibiting its cell protective activities. Here, we report that ATO in combination with Dasatinib exerts significant synergistic pro‐apoptotic effects, mainly through activating the IRE1/JNK/PUMA cell apoptosis pathway, while neutralizing the ATF4 cell survival pathway.

Strikingly, we further found that ATO plus Dasatinib synergistically repress some genes whose expressions are associated with shorter survival probabilities in ALL patients. These findings suggest that ATO and Dasatinib combined together should be a promising therapeutic strategy for Ph^+^ ALL.

## Materials and methods

### Cell culture

Human Ph^+^ ALL cell lines SUP‐B15 (TKI‐resistant) and TOM‐1(TKI‐sensitive) were cultured in IMDM (Gibco, Carlsbad, CA, USA) supplemented with 20% foetal bovine serum (Gibco) at 37°C in a 5% CO_2_ atmosphere.

### Reagents and antibodies

DMSO was purchased from Sigma‐Aldrich (St. Louis, MO, USA). Dasatinib was purchased from Cell Signaling Technology (CST, Danvers, MA, USA) and dissolved in DMSO. ATO was purchased from the First Affiliated Hospital of Harbin Medical University. c‐ABL1, Bcl‐2, Mcl‐1, Noxa and p53 antibodies were purchased from Santa Cruz Biotechnology (Santa Cruz, CA, USA); β‐actin antibody was bought from Sigma‐Aldrich; ERK, p‐ERK, STAT5, p‐STAT5, PI3K, p‐PI3K, AKT, p‐AKT, caspase‐9, caspase‐3, PARP, XIAP, Survivin, Bcl‐w, Bik, Bak, Bad, PUMA, Bid, p21, JNK, p‐JNK, p‐ATF‐2, p‐JUN, IRE1, ASK1 and ATF4 antibodies were purchased from CST; TRAF2 antibody was purchased from ABclonal Biotechnology (Wuhan, Hubei, China); PML and ATF6 were purchased from Abcam (Cambridge, MA, USA).

### Cell viability assay

5 × 10^4^ cells were seeded in 96‐well plates. After treatment with ATO and/or Dasatinib for 24, 36 or 48 hrs, 10 μl CCK8 (Dojindo Laboratories, Kumamoto, Japan) was added to each well and incubated for another 1.5–3 hrs at 37°C. Absorbance of the samples was measured against a background control at 450 nm with a reference wavelength of 600 nm.

### Analysis for synergy

The Combination Index (CI) of ATO and Dasatinib was calculated using Calcusyn^®^ (Great Shelford, Cambridge, UK). CI < 1, CI = 1 and CI > 1 indicated synergism, additive and antagonism effect, respectively 13.

### Western blot analysis

Total cell lysates were extracted using RIPA lysis buffer containing 1× cocktail inhibitor and 1 mM PMSF. Equal amounts of total proteins were separated by 7.5–15% SDS‐PAGEs and transferred to PVDF membranes. After being blocked with 5% BSA for 1 hr at room temperature (RT), each membrane was incubated overnight at 4°C with the primary antibodies. After washing three times with 1 × TBST, each membrane was incubated with HRP‐goat‐anti‐mouse IgG or anti‐rabbit IgG (CST) as secondary antibody for 1 hr at 4°C. The expressions of the proteins of interest were detected by enhanced chemiluminescence kits (Millipore, Billerica, MA, USA).

### Co‐immunoprecipitation (Co‐IP)

Total cell lysates were extracted using lysis buffer [50 mM HEPES (pH 7.4), 150 mM NaCl, 2 mM CaCl2, 0.5% NP‐40, 5% glycerol] containing 1× cocktail inhibitor and 1 mM PMSF for 30 min on ice and centrifuged at 12,000× g for 15 min at 4°C. The supernatants were collected and incubated with protein A/G agarose beads (Santa Cruz) for 2 hrs at 4°C for pre‐clear and further incubated with anti‐IRE1 antibodies overnight at 4°C with constant rotation for immunoprecipitation. Subsequently, protein A/G agarose beads were added, and the mixture was incubated for another 2 hrs at 4°C. Then, the beads were washed six times with washing buffer [50 mM HEPES (pH 7.4), 150 mM NaCl, 2 mM CaCl2, 0.5% NP‐40], followed by boiling with 25 μl 2 × SDS‐PAGE loading buffer for 5 min. Then, the samples were centrifuged at 12,000× g for 30 seconds (s) with the supernatants collected and stored at −80°C.

### Real‐time PCR

Total RNA was extracted using TRIzol reagent (Invitrogen, Carlsbad, CA, USA), and the cDNA was obtained using Hieff™ First Strand cDNA Synthesis Kit (YEASEN, Shanghai, China). Real‐time PCR assay was performed using HieffTM qPCR SYBR^®^ Green Master Mix (Low Rox Plus) (YEASEN). The primers were as follows: GAPDH (Forward: GAAGGTGAAGGTCGGAGTC, Reverse: GAAGATGGTGATGGGATTTC); AGPS (Forward: TTAGTGGCATGGGTTTACCAAC, Reverse: CTCGATCATCTGCCTCTTGTG); CDK6 (Forward: CCAGATGGCTCTAACCTCAGT, Reverse: AACTTCCACGAAAAAGAGGCTT); ATAD2 (Forward: AGGCTCCATTGGAAAAACCT, Reverse: CCTGCGGAAGATAATCGGTA); ARPP19 (Forward: AAGGAAACGGTTGCAGAAAGG, Reverse: GGAGTGGGAATGTGGTCACC); SC4MOL (Forward: TGCCTTTGATTTGTGGAACCTATT, Reverse: GTGCCAAGTATCTTCAATGACTGC); ACSL1 (Forward: CGACGAGCCCTTGGTGTATTT, Reverse: GGTTTCCGAGAGCCTAAACAA); IGFBP7 (Forward: CGAGCAAGGTCCTTCCATAGT, Reverse: GGTGTCGGGATTCCGATGAC); STARD4 (Forward: AGAAGGGCTTTTATCTTGTGGA, Reverse: CCAACCACAGGGATGGTTAT). Amplifications were performed in a 7500 Real‐time PCR System (Applied Biosystems, Foster City, CA, USA).

### Preparation of lentiviral expression vectors

pLVX‐shRNA2 vector was purchased from Clontech Laboratories (Mountain View, CA, USA). Recombinant lentiviral shPUMA (with a target sequence of 5′‐CGTGTGACCACTGGCATTCAT‐3′) 14, shIRE1‐1 (with a target sequence of 5′‐GAGAAGATGATTGCGATGGAT‐3′), shIRE1‐2 (with a target sequence of 5′‐GAAATACTCTACCAGCCTCTA‐3′) 15, shATF4‐1 (with a target sequence of 5′‐GCCTAGGTCTCTTAGATGATT‐3′) and shATF4‐2 (with a target sequence of 5′‐GCCAAGCACTTCAAACCTCAT‐3′) were constructed according to the manufacturer's instructions.

pLVX‐IRES‐Puro vector was also purchased from Clontech Laboratories. cDNA of JNK1‐a2 and JNK1‐b1 was amplified by PCR and cloned into pLVX‐IRES‐Puro vectors. The kinase mutants of JNK1‐a2 and JNK1‐b1 (JNK1‐a2‐M and JNK1‐b1‐M) were constructed by a Fast Mutagenesis System (TransGen Biotech, Beijing, China). The primers were as follows: Forward 5′‐ACGAGTTTTATGATGGCGCCTGCTGTAGTGACT‐3′; Reverse 5′‐GCAGGCGCCATCATAAAACTCGTTCCTGCAGTC‐3′.

### Construction of knockdown or overexpression cell lines

The retroviral supernatants from HEK293T cells produced by co‐transfection of pLVX‐shRNA2/pLVX‐IRES‐Puro, pMD2.G (Addgene, Cambridge, MA, USA) and psPAX2 (Addgene) were filtered with a 0.22 μm Sterile Milles Filter Unit (Millipore). The filtrates were then used to re‐suspend SUP‐B15 cells, centrifuged at 1200× g for 90 min and incubated for another 24 hrs at 37°C. Cells were infected twice and selected with puromycin (Sigma‐Aldrich, from 0.5 μg/ml to 4 μg/mL).

### Apoptosis assays

1 × 10^5^ cells were washed with 1 × PBS and re‐suspended in 200 μL staining solution containing 2 μL of Annexin V–FITC and 2 μL of 20 μg/mL PI (FITC‐Annexin V staining kit, Clontech). Flow cytometry was performed to analyse 1 × 10^4^ cells. All data were collected, stored and analysed by FlowJo (TreeStar).

### Statistical analyses

Data are expressed as the mean ± S.E.M. Significance was calculated using Student's t‐test. A two‐tailed value of *P* < 0.05 was considered to be significant.

## Results

### Combination of ATO and Dasatinib exhibit significant synergistic adverse effects on the viability of Ph^+^ ALL cell lines

**Figure 1:**
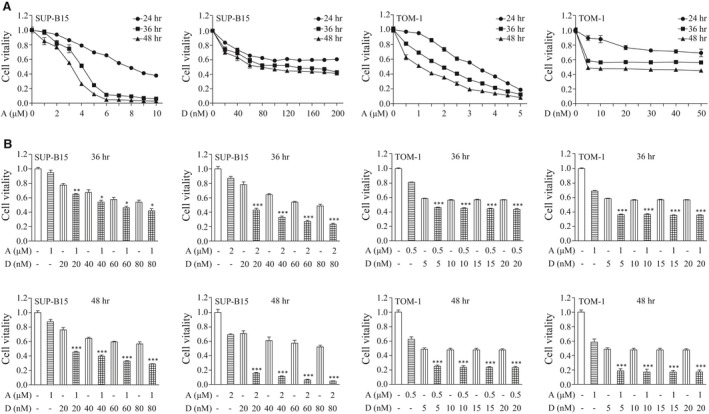
Effect on cell vitality of a combination of ATO and Dasatinib on Ph^+^ ALL cell lines. (**A**) Concentration‐dependent and time course‐dependent effects of ATO or Dasatinib on SUP‐B15 and TOM‐1 cells. Cells were treated with the indicated concentrations of ATO or Dasatinib for the indicated time courses. Cell viability was measured by CCK‐8. (**B**) Synergistic effects of ATO and Dasatinib on SUP‐B15 and TOM‐1 cells. Cell viability was analysed after a treatment with ATO and/or Dasatinib at the indicated concentrations. Each bar represents the mean ± S.E.M, *n* = 3. *P < 0.05; **P < 0.01; ***P < 0.001 *versus* ATO, Dasatinib and control group. A, ATO; D, Dasatinib.

To detect the effects of ATO and Dasatinib on cell viability, we firstly treated Ph^+^ ALL cell lines SUP‐B15 and TOM‐1 with ATO or Dasatinib for 24, 36 and 48 hrs, respectively (Fig. 1A). We found that TOM‐1 cells were more sensitive to Dasatinib than SUP‐B15 cells. These results are in agreement with a previous report showing that SUP‐B15 cells were resistant to TKI, while TOM‐1 cells were not 2. Then, increasing concentrations of ATO and Dasatinib were used to treat SUP‐B15 and TOM‐1 cells for different time periods (Fig. 1B). As a result, we observed that ATO plus Dasatinib was more effective in affecting cell viability than each drug used alone in both SUP‐B15 and TOM‐1 cells, treated during either 36 or 48 hrs. Also, the Combination Index (CI) was less than one under all the used drug concentrations, suggesting that ATO and Dasatinib have synergistic effects (Table S1).

### ATO along with Dasatinib in Ph^+^ ALL cell lines neither degrade BCR‐ABL1 nor synergistically inhibit the three main downstream pathways of BCR‐ABL1

Previous research demonstrated that ATO at the concentration of 1 or 2 μΜ induces the degradation of BCR‐ABL1 in CML‐blast crisis cell line, K562 16. We indeed found that a higher concentration of ATO (over 4 μΜ) could down‐regulate BCR‐ABL1 in SUP‐B15 (Fig. S1). However, we also observed that a lower concentration of ATO, used alone or combined with Dasatinib, has no effect on BCR‐ABL1 degradation (Figs S1 and S2). In comparison, the expressions of PML (a classical target protein of ATO) in SUP‐B15 or TOM‐1 and of BCR‐ABL1 in K562 were both remarkably down‐regulated by lower concentrations of ATO (Fig. S2). This observation suggested that the synergistic effects found here on cell viability using ATO and Dasatinib are mainly independent from the degradation of BCR‐ABL1.

The oncogenic activity of BCR‐ABL1 relies on its three main downstream pathways: Ras/MAPK (ERK), JAK/STAT5 and PI3K/AKT. Here, we observed that JAK/STAT5 and ERK are inhibited by Dasatinib, whereas PI3K/AKT is not. More importantly, no synergistic inhibitory effect of ATO and Dasatinib was detected on the activity of ERK, JAK/STAT5 or PI3K/AKT (Fig. S3). This suggested that the synergistic effects of ATO and Dasatinib on cell viability did not rely much on BCR‐ABL1 and on its three main downstream pathways.

### ATO and Dasatinib used in combination induce a higher level of apoptosis in Ph^+^ ALL cell lines than ATO or Dasatinib used alone

**Figure 2:**
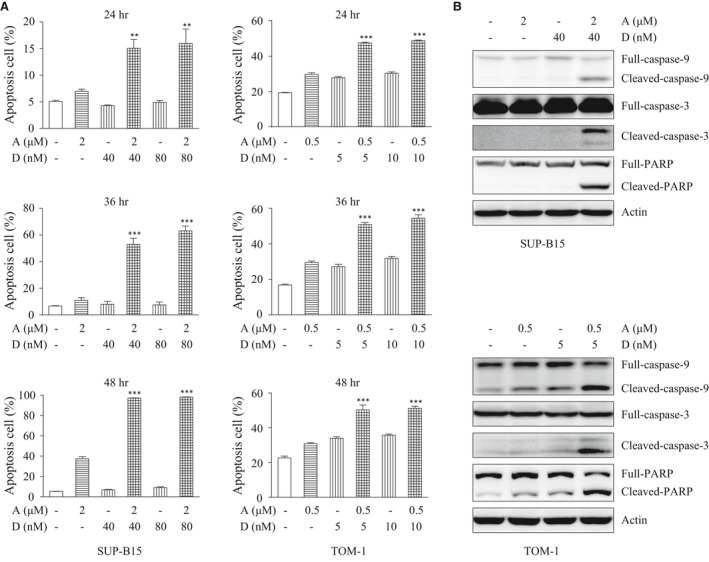
ATO combined with Dasatinib induces apoptosis. (**A**) SUP‐B15 and TOM‐1 cells were treated with the indicated concentrations of ATO and/or Dasatinib for the indicated time courses. Apoptosis was measured by flow cytometry with FITC‐Annexin‐V/PI staining. (**B**) Western blot detecting caspase‐9,3 and PARP in SUP‐B15 and TOM‐1 cells treated with ATO and/or Dasatinib at the indicated concentrations for 24 hrs. Each bar represents the mean ± S.E.M, *n* = 3. **P < 0.01; ***P < 0.001 *versus* ATO, Dasatinib and control group.

To clarify the mechanism underlying the synergistic actions of ATO and Dasatinib, we measured cell apoptosis after ATO and/or Dasatinib treatments. Our findings were that: (*i*) the single‐agent Dasatinib induces apoptosis in TOM‐1 cells but not in SUP‐B15 cells, further confirming that SUP‐B15 cells are resistant to TKI; (*ii*) ATO induces apoptosis both in SUP‐B15 and TOM‐1 cells; and more importantly (*iii*) the combination of ATO and Dasatinib induces a much higher level of apoptosis (Fig. 2A). In addition, analysing the cleavage of apoptosis‐related proteins caspase‐9, 3 and PARP suggests that the combination of ATO plus Dasatinib induces apoptosis through the caspase pathway (Fig. 2B).

### ATO and Dasatinib combined together strongly up‐regulate the expression of the pro‐apoptotic protein PUMA

**Figure 3:**
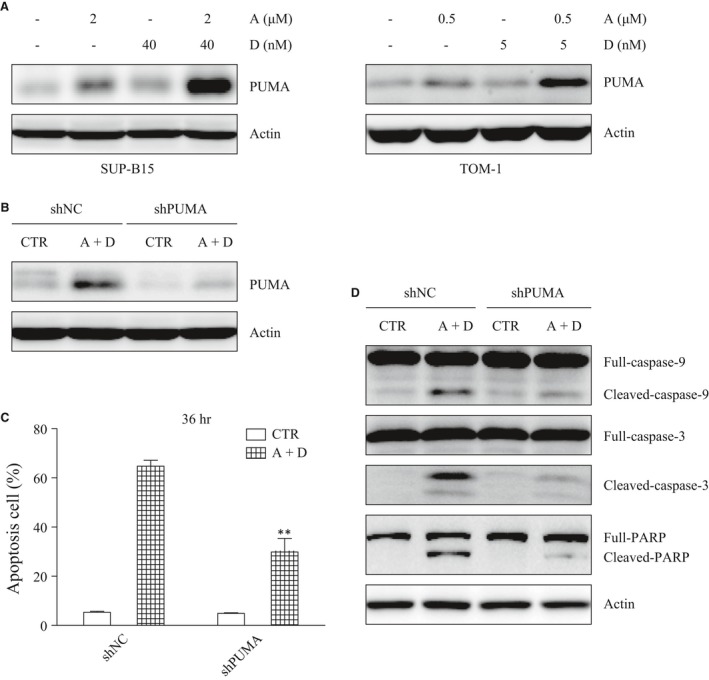
The knockdown of PUMA inhibits the apoptosis induced by ATO combined with Dasatinib. (**A**) The expression of PUMA was detected by Western blot after a 24‐hr treatment with ATO and/or Dasatinib. (**B**) SUP‐B15 cells were stably transfected with control or PUMA shRNA. Stably transfected cells were treated with ATO (2 μΜ) combined with Dasatinib (40 nM) for 24 hrs. (**C**) Apoptosis was measured in the stably transfected cells with or without ATO (2 μΜ) and Dasatinib (40 nM) treatment. (**D**) Western blot detecting caspase‐9,3 and PARP in the stably transfected cells after a 24‐hr treatment with ATO (2 μΜ) and Dasatinib (40 nM). Bars represent the mean ± S.E.M, *n* = 3. **P < 0.01 *versus* shNC (A+D) group.

To further elucidate how ATO plus Dasatinib triggered apoptosis, we detected the expression of several apoptosis‐related proteins of the BCL‐2, IAP and Flip families. The most important change was the expression of PUMA, which was up‐regulated by the single‐agent ATO and increased dramatically after the ATO plus Dasatinib combination treatment (Figs 3A and S4). Short hairpin RNAs (shRNA) were then used to down‐regulate PUMA in SUP‐B15 cells (Fig. 3B). Consequently, in PUMA knock‐down cells, we observed a significant decrease in apoptosis, which was associated with lower levels of activated caspase‐9, 3 and PARP (Figs 3C and D). Taken together, these findings demonstrate that the apoptosis induced by ATO plus Dasatinib is PUMA‐dependent.

### The activation of the JNK pathway is responsible for PUMA up‐regulation as well as for ATO plus Dasatinib‐induced apoptosis

**Figure 4:**
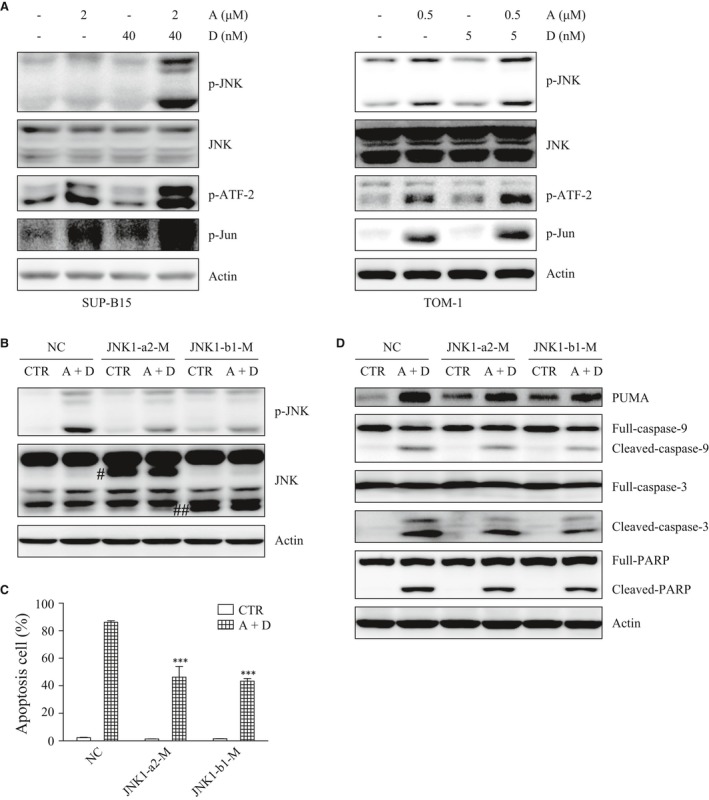
The activation of the JNK pathway is responsible for PUMA up‐regulation and apoptosis induced by ATO combined with Dasatinib. (**A**) JNK, p‐JNK, p‐ATF‐2 and p‐Jun were detected by Western blot after ATO and/or Dasatinib treatment for 24 hrs. (**B**) SUP‐B15 cells were stably transfected with NC, JNK1‐a2‐M or JNK1‐b1‐M. Stably transfected cells were treated with ATO (2 μΜ) combined with Dasatinib (40 nM) for 24 hrs. The expressions of JNK and p‐JNK were detected by Western blot. (**C**) Stably transfected cells were treated with ATO (2 μΜ) combined with Dasatinib (40 nM) for 48 hrs, and apoptosis was measured by flow cytometry. (**D**) Stably transfected cells were treated with ATO (2 μΜ) combined with Dasatinib (40 nM) for 24 hrs. The expressions of PUMA, caspase‐9, 3 and PARP were detected by Western blot. ^#^JNK1‐a2‐M; ^##^JNK1‐b1‐M. Each bar represented the mean ± S.E.M, *n* = 3. ***P < 0.001 *versus* NC (A+D) group.

PUMA is known to be regulated by p53, c‐myc, JNK and other factors. In this study, p53 and p21, a main downstream target of p53, were down‐regulated by Dasatinib, both in SUP‐B15 and TOM‐1 cells. However, after the ATO plus Dasatinib combination treatment, the expressions of p53 and p21 were down‐regulated similarly as after single‐agent Dasatinib in TOM‐1 cells, and no significant differences were detected in SUP‐B15 cells. In addition, although c‐myc was down‐regulated by ATO and/or Dasatinib, its regulation modes were quite different from PUMA (Fig. S5). On the contrary, JNK was significantly activated in cells receiving the combination treatment group, compared to each drug used alone. Additionally, JUN and ATF‐2, the two main downstream targets of JNK, were also activated by the combination of ATO and Dasatinib (Fig. 4A). These results suggest that PUMA is activated by JNK in our model system.

More interestingly, we also found that PUMA, JUN and ATF‐2 are activated by a single‐agent ATO in both cell lines, whereas JNK was only activated in TOM‐1, and not in SUP‐B15. To explain this observation, SUP‐B15 cells were treated with ATO for 0, 3, 6, 12 and 24 hrs, respectively. We observed the following: (*i*) JNK was activated after 3 hrs of ATO treatment and then was rapidly inactivated; (*ii*) ATF‐2 was activated by ATO with a peak at 6 hrs; and (*iii*) PUMA began to be activated after 6 hrs of ATO treatment with a peak at 12 hrs (Fig. S6). These results implied that the JNK pathway was only activated temporarily by the single‐agent ATO in SUP‐B15. Nevertheless, under both ATO and Dasatinib treatments, the JNK pathway was stably activated.

As previously reported, the transient activation of JNK is involved in cell survival, while persistent activation induced cell apoptosis 17. Next, we explored whether the activation of JNK played an important role in PUMA up‐regulation and apoptosis in SUP‐B15. To this aim, we over‐expressed a kinase‐dead mutant of JNK that acts as a dominant negative to inactivate the endogenous JNK. There are three independent JNK genes, namely, JNK1, 2 and 3. JNK1 and JNK2 are widely expressed, while JNK3′s expression is predominantly restricted to the brain, heart and testis. Studies suggest that the activation of JNK1 may induce apoptosis, whereas JNK2 inhibits it 18, 19. At least four alternatively spliced transcript variants of JNK1 exist, including two short JNK1 isoforms (JNK1‐a1 and JNK1‐b1) and two long JNK1 isoforms (JNK1‐a2 and JNK1‐b2). In this study, kinase‐mutated JNK1‐a2 (JNK1‐a2‐M) and JNK1‐b1 (JNK1‐b1‐M) were stably transfected into SUP‐B15 cells. As a result, the JNK activity was reduced significantly in both JNK1‐a2‐M and JNK1‐b1‐M transfected cells compared with control cells (NC) (Fig. 4B). More remarkably, apoptosis was decreased in both JNK1‐a2‐M and JNK1‐b1‐M cells, as well as the expression of PUMA and the cleavages of caspase‐9, 3, PARP (Figs. 4C and D). Collectively, these results indicated that JNK1 plays a key role in PUMA up‐regulation and in the apoptosis induced by ATO plus Dasatinib.

### IRE1 activates JNK and plays a pivotal role in the combination of ATO and Dasatinib treatment effects

**Figure 5:**
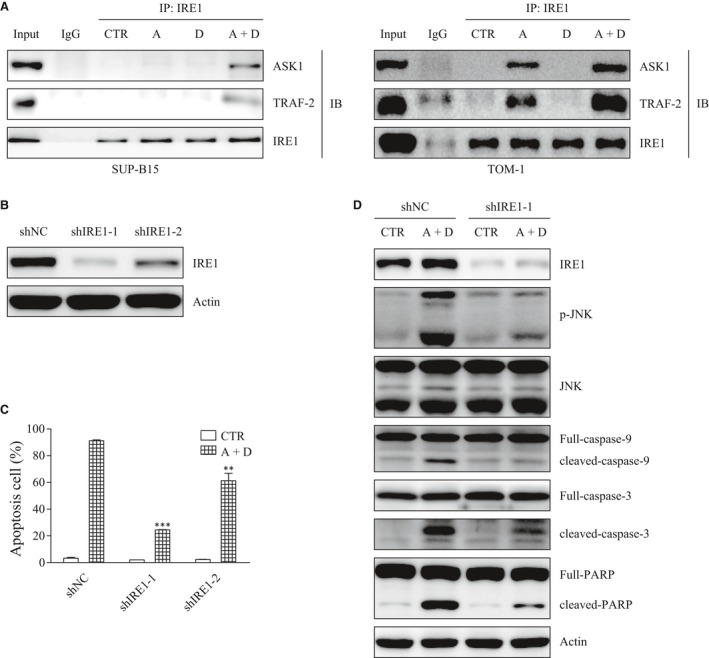
Knockdown of IRE1 blocks the apoptosis induced by ATO combined with Dasatinib. (**A**) SUP‐B15 and TOM‐1 cells were treated with ATO and/or Dasatinib for 24 hrs. Cell lysates were immunoprecipitated using anti‐IRE1 antibody or normal rabbit IgG. ASK1, TRAF2 and IRE1 were detected by Western blot. (**B**) SUP‐B15 cells were stably transfected with shNC, shIRE1‐1 and shIRE1‐2. (**C**) Apoptosis was measured in the stably transfected cells with or without a 24‐hr treatment with ATO (2 μΜ) and Dasatinib (40 nM). (**D**) IRE1, p‐JNK, JNK, caspase‐9, 3 and PARP were detected by Western blot in the stably transfected cells after a 24‐hr treatment with ATO (2 μΜ) and Dasatinib (40 nM). Bars represent the mean ± S.E.M, *n* = 3. **P < 0.01; ***P < 0.001 *versus* shNC (A+D) group.

JNK is known to be activated by ASK1/2, which is recruited and activated by IRE1 through its binding to TRAF2 during ER stress 20. We discovered a clear interaction between IRE1 and both ASK1 and TRAF2 in the cells treated by the combination ATO plus Dasatinib in SUP‐B15 as well as in TOM‐1 cells (Fig. 5A). shRNAs were then used to stably knock‐down IRE1 (shNC, shIRE1‐1 and shIRE1‐2) in SUP‐B15 (Fig. 5B). We found that the knockdown of IRE1 induces a significant decrease of the apoptosis triggered by ATO plus Dasatinib and prevents the activation of JNK and the cleavage of caspase‐9,3 and PARP (Figs. 5C and D). Collectively, these results suggested that the combination of ATO and Dasatinib activates JNK to induce apoptosis through the UPR axis IRE1.

### ATF4 plays a protective role in cell apoptosis

**Figure 6:**
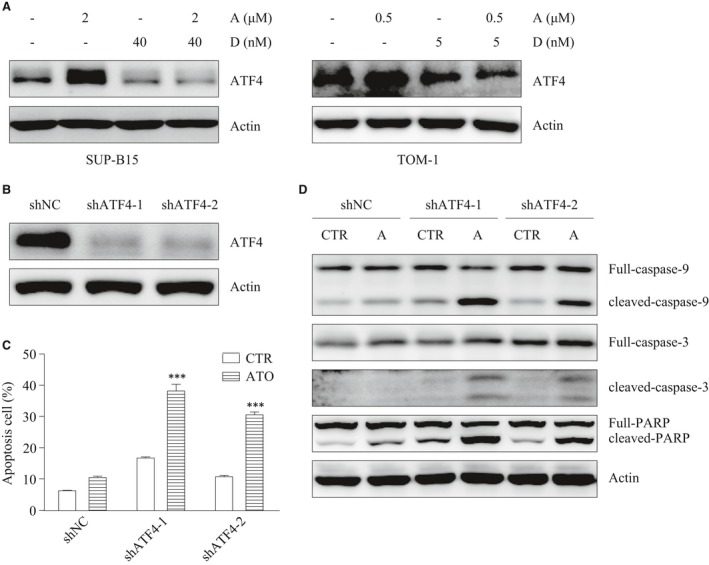
The down‐regulation of ATF4 increases cells sensitivity to ATO. (**A**) ATF4 was detected by Western blot in SUP‐B15 and TOM‐1 cells after a 24‐hr treatment with ATO and/or Dasatinib. (**B**) SUP‐B15 cells were stably transfected with shNC, shATF4‐1 and shATF4‐2. (**C**) Apoptosis was measured in the stably transfected cells with or without ATO (2 μΜ) treatment for 24 hrs. (**D**) caspase‐9, 3 and PARP were detected by Western blot in the stably transfected cells after ATO (2 μΜ) treatment for 24 hrs. Bars represent the mean ± S.E.M, *n* = 3. ***P < 0.001 *versus* shNC ATO group.

As we mentioned above, in addition to IRE1/XBP1s, there are two other main UPR axes: PERK/eIF2α/ATF4 and ATF6, the expression of which we also detected after ATO and/or Dasatinib treatment. We observed that ATF4 was up‐regulated by ATO, but showed no increase when ATO was combined with Dasatinib in both SUP‐B15 and TOM‐1 cell lines (Fig. 6A). Under the same conditions, ATF6 did not change significantly (data not shown). As ATF4 can either promote cell survival or induce cell apoptosis, we decided to further explore the exact role ATF4 in our model systems. shRNAs were used to stably knock‐down ATF4 (shNC, shATF4‐1 and shATF4‐2) in SUP‐B15 cells (Fig. 6B). The knockdown of ATF4 significantly increased apoptosis, as well as the cleavage of caspase‐9,3 and PARP triggered by ATO (Fig. 6C and D). This suggested that, although ATO may protect cells from apoptosis by up‐regulating ATF4, this protective effect could be abolished when ATO and Dasatinib are used in combination.

Collectively, these results demonstrate that the combination of ATO and Dasatinib synergistically induces cell apoptosis in Ph^+^ ALL cell lines both by activating the IRE1‐JNK‐PUMA apoptosis pathway and abolishing the ATF4‐induced cell survival pathway.

### ATO and Dasatinib synergistically regulate genes associated with shorter survival in ALL patients

Previously, we defined a six‐gene signature identifying aggressive and treatment‐resistant ALL in children and adults (in our work this group of ALL was named ‘P3’). This situation enabled us to perform a supervised transcriptomic analysis to look for genes differentially expressed between these aggressive ‘P3’ ALL and ALLs associated with higher survival probabilities that we named “P1&2” 21 (Fig. S7A). Moreover, following this approach we discovered that a subset of ‘P1&2’ ALL displayed a general transcriptome which was similar to the ‘P3’ group of aggressive ALL, although their survival probabilities remained higher. This subset of ALL was called P1&2 ‘P3‐like’ (Fig. S7B). Based on the hypothesis that the differences in gene expression between P3 and P1&2 ‘P3‐like’ ALL should identify genes whose activation would be directly related to the aggressiveness of the ALL, we compared the transcriptomes of these two subsets of ALL. This approach led to the identification of a group of 27 genes, which are particularly up‐regulated in P3 ALL (Table S2) and are hence good candidates to be drivers of the aggressive nature of these malignancies.

**Figure 7:**
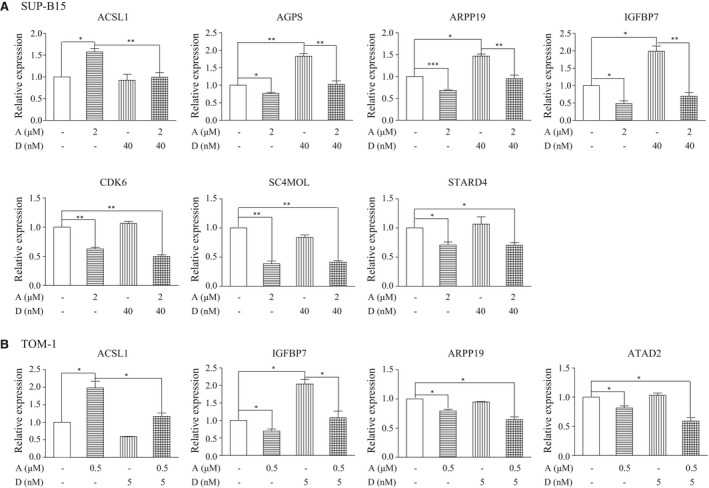
ATO combined with Dasatinib synergistically regulates some genes associated with shorter survival in ALL. (**A**) ACSL1, AGPS, ARPP19, IGFBP7, CDK6, SC4MOL and STARD4 were detected by real‐time PCR in SUP‐B15 cells after a 24‐hr treatment with ATO and/or Dasatinib. (**B**) ACSL1, IGFBP7, ARPP19 and ATAD2 were detected by real‐time PCR in TOM‐1 cells after a 24‐hr treatment with ATO and/or Dasatinib. Bars represent the mean ± S.E.M, *n* = 3. *P < 0.05; **P < 0.01; ***P < 0.001.

Here, we took advantage of these findings to monitor the expression of these 27 genes after ATO and/or Dasatinib treatments, with the findings that: (*i*) in TKI‐resistant SUP‐B15 cells, ACSL1 was up‐regulated by ATO, but reversed by ATO plus Dasatinib; AGPS, ARPP19 and IGFBP7 were up‐regulated by Dasatinib, but reversed by ATO plus Dasatinib; CDK6, SC4MOL and STARD4 were down‐regulated by ATO or ATO plus Dasatinib (Fig. 7A). (*ii*) in TKI‐sensitive TOM‐1 cells, ACSL1 and IGFBP7 were similarly regulated as in SUP‐B15 cells; ARPP19 and ATAD2 were down‐regulated by ATO and further down‐regulated by ATO plus Dasatinib, but not by single‐agent Dasatinib (Fig. 7B).

Taken together, these results lead to the following conclusions: (*i*) ATO and Dasatinib could synergistically down‐regulate some genes associated with particularly poor prognosis in ALL; (*ii*) the inability of Dasatinib to down‐regulate ATAD2 in TKI‐resistant SUP‐B15 cells may partly account for TKI resistance, suggesting that specifically targeting ATAD2 may improve the outcome of TKI‐resistant patients; (*iii*) the regulation of AGPS, CDK6, SC4MOL and STARD4 by ATO plus Dasatinib in SUP‐B15 cells may also account for the observed strong synergistic effect of ATO and Dasatinib used in combination in treating TKI‐resistant Ph^+^ ALL.

## Discussion

TKI resistance is a life‐threatening problem in Ph^+^ ALL patients. In the present study, we demonstrate that a combination of ATO and Dasatinib is extremely effective in targeting Ph^+^ ALL cells.

We demonstrate that ATO combined with Dasatinib trigger apoptosis by simultaneously activating the IRE1/JNK/PUMA apoptosis pathway and inhibiting the ATF4 survival pathway. IRE1 is an ER transmembrane protein possessing both Ser/Thr kinase and endoribonuclease (RNase) activities. Upon ER stress, the oligomerized IRE1 becomes autophosphorylated, which activates its RNase activity leading to the cleavage of XBP1 mRNA. Spliced XBP1 encodes transcriptional factors, XBP1s, which enhance the ER protein folding capacity and ER‐induced protein degradation to alleviate ER stress. However, when ER stress is unrecoverable, IRE1 also causes endonucleolytic decay of some ER‐localized mRNAs, such as GRP78, to trigger apoptosis 22, 23. Another study further confirmed that pseudokinase‐activated IRE1 could also cleave the XBP1 mRNA, but not trigger apoptosis, suggesting that phosphorylation of IRE1 is crucial for IRE1‐induced apoptosis 22. This is in agreement with the finding that IRE1 can form a complex with TRAF2 and ASK1, which activates ASK1/JNK signalling pathway to trigger cell apoptosis 24. In the present work, we also observe that ATO plus Dasatinib promotes the formation of IRE1‐TRAF2‐ASK1 complex to trigger apoptosis. However, the relationship between IRE1‐dependent mRNA decay and activation of the ASK1/JNK apoptosis pathway needs to be further investigated.

ATF4 is a cAMP response element‐binding transcription factor. Translation of ATF4 is induced by stress signals including ER stress, amino acid deprivation, anoxia/hypoxia and oxidative stress through phosphorylation of eIF2α. ATF4 protects cells against adverse conditions through activating genes whose product function is involved in amino acid import and metabolism, oxidative stress response and protein secretion 25. In addition, ATF4 can directly target Mcl‐1, an anti‐apoptosis member of the Bcl‐2 family, or genes implicated in autophagy, to protect cells from apoptosis 26, 27. However, ATF4 can also trigger apoptosis through targeting genes involved in protein synthesis, which cause ATP depletion and oxidative stress 28.

The precise mechanism by which ATF4 switches from anti‐apoptosis to pro‐apoptosis activities has not been studied. Armstrong reported that transcriptional activation of ATF4 mediates ER stress‐induced cell death 29. In the present work, we found that ATO was up‐regulated ATF4 at the protein level, but not at the mRNA level (data not shown), showing that the transcriptional activity of ATF4 was not affected by ATO. Meanwhile, we also observed that ATF4 protected cells from apoptosis. Thus, it seems that some transcription factors, by regulating the transcriptional activation of ATF4, may work as ‘switches’ between anti‐apoptosis and pro‐apoptosis, a hypothesis that needs to be further demonstrated.

The limitation of this work is that we did not focus on the T315I mutation of BCR‐ABL1, one of the main mechanisms of resistance to TKIs. Future directions include the construction of a T315I‐mutated SUP‐B15 cell line, using the CRISPR/CAS9 gene editing technology, in which we would test the effectiveness of ATO plus Dasatinib. However, it is possible to predict that the combination of ATO and Dasatinib would not be effective in treating T315I mutation, as Dasatinib does not work on T315I mutation. A third‐generation TKI, Ponatinib, which can inhibit the activity of T315I mutated BCR‐ABL1, might be alternatively combined with ATO.

Translating our findings into the clinics will also require testing the safeness and effectiveness of ATO plus Dasatinib in pre‐clinical studies. We are presently establishing Ph^+^ ALL patient‐derived xenograft (PDX) models. If ATO plus Dasatinib works well in these animal models, it will set a basis to explore this combination in clinical trials on Ph^+^ ALL patients with relapse and/or resistance to current treatments. However, whether this combination strategy could be used for maintenance (after multi‐agent chemotherapy) or even up‐front treatments, needs to be evaluated, depending on the outcome of the clinical trials.

In conclusion, we have identified a synergistic anti‐leukaemia effect of ATO and Dasatinib used together in two Ph^+^ ALL cell lines. The underlying mechanism involves the promotion of cell apoptosis through IRE1 and the concomitant neutralization of the ATF4‐dependent cell survival. As ATO and Dasatinib are both approved by the US Food and Drug Administration, in the near future, these findings should lead to pre‐clinical and clinical studies to determine whether the combination is safe and effective in Ph^+^ ALL patients.

## Conflicts of interest

The authors confirm that there are no conflicts of interest.

## Supporting information


**Figure S1.** Effects of single‐agent ATO on BCR‐ABL1.
**Figure S2.** Effects of ATO and/or Dasatinib on BCR‐ABL1 in SUP‐B15, TOM‐1 or K562 cell lines.
**Figure S3.** Effects of ATO and/or Dasatinib on the three main downstream signaling pathways of BCR‐ABL1.
**Figure S4.** The expression of proteins of the BCL‐2, IAP and Flip families were detected by Western blots after ATO and/or Dasatinib 24 hrs treatment in SUP‐B15 cells.
**Figure S5.** p53, p21 and c‐myc were detected by Western blot after a 24 hrs treatment with ATO and/or Dasatinib.
**Figure S6.** JNK, p‐JNK, p‐ATF‐2 and PUMA were detected by Western blot after a treatment with ATO (2 μΜ) for the indicated time courses in SUP‐B15 cells.
**Figure S7.** Strategy for the identification of 27 genes whose activation would be directly related to the aggressiveness of the ALL.
**Table S1.** Combination Index (CI) of ATO and Dasatinib in SUP‐B15 and TOM‐1 cells.
**Table S2.** Genes up‐regulated in P3 *versus* P1&2 “P3‐like”.
