# Supplementary material for: Combination of arsenic trioxide and Dasatinib: a new strategy to treat Philadelphia chromosome‐positive acute lymphoblastic leukaemia
Source: J Cell Mol Med. 2017 Dec 20;22(3):1614–26. doi: 10.1111/jcmm.13436 (PMC5824394; doi:10.1111/jcmm.13436)
Supplement: Supplementary file 1 — Figure S1. Effects of single‐agent ATO on BCR‐ABL1. Figure S2. Effects of ATO and/or Dasatinib on BCR‐ABL1 in SUP‐B15, TOM‐1 or K562 cell lines. Figure S3. Effects of ATO and/or Dasatinib on the three main downstream signaling pathways of BCR‐ABL1. Figure S4. The expression of proteins of the BCL‐2, IAP and Flip families were detected by Western blots after ATO and/or Dasatinib 24 hrs treatment in SUP‐B15 cells. Figure S5. p53, p21 and c‐myc were detected by Western blot after a 24 hrs treatment with ATO and/or Dasatinib. Figure S6. JNK, p‐JNK, p‐ATF‐2 and PUMA were detected by Western blot after a treatment with ATO (2 μΜ) for the indicated time courses in SUP‐B15 cells. Figure S7. Strategy for the identification of 27 genes whose activation would be directly related to the aggressiveness of the ALL. Table S1. Combination Index (CI) of ATO and Dasatinib in SUP‐B15 and TOM‐1 cells. Table S2. Genes up‐regulated in P3 versus P1&2 “P3‐like”. [file JCMM-22-1614-s001.pdf]

Combination of Arsenic trioxide and Dasatinib: A New strategy to Treat Philadelphia chromosome positive acute lymphoblastic leukemia

Supplementary data

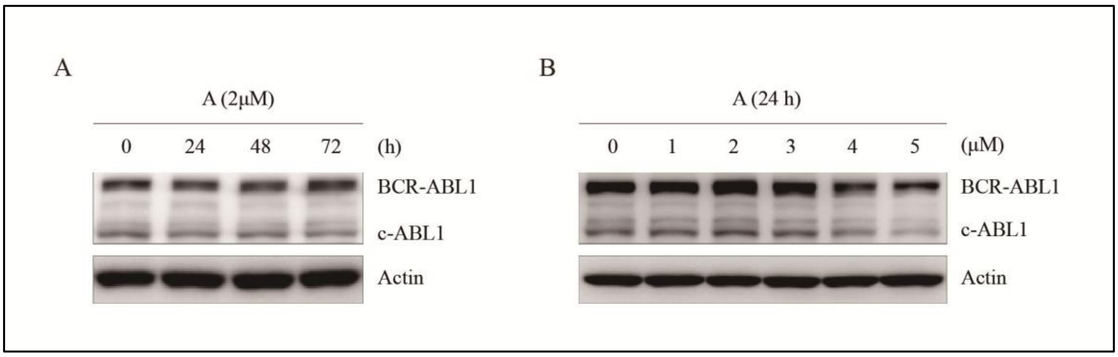

Fig. S1 Effects of single-agent ATO on BCR-ABL1. **(A)** SUP-B15 cells were treated with ATO for the indicated time courses. **(B)** SUP-B15 cells were treated with the indicated concentrations of ATO for 24 h. The expression of BCR-ABL1 (c-ABL1) was determined by Western blot. A, ATO.

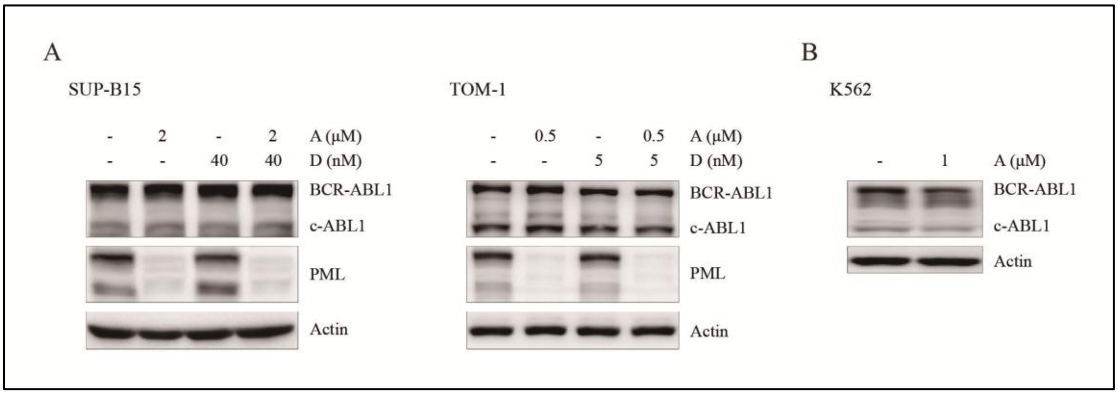

Fig. S2 Effects of ATO and/or Dasatinib on BCR-ABL1 in SUP-B15, TOM-1 or K562 cell lines. **(A)** SUP-B15 and TOM-1 cells were treated with ATO and/or Dasatinib for 24 h. The expression of BCR-ABL1 (c-ABL1) and PML were determined by Western blot. **(B)** K562 cells were treated with ATO for 24 h. The expression of BCR-ABL1 (c-ABL1) was determined by Western blot. D, Dasatinib.

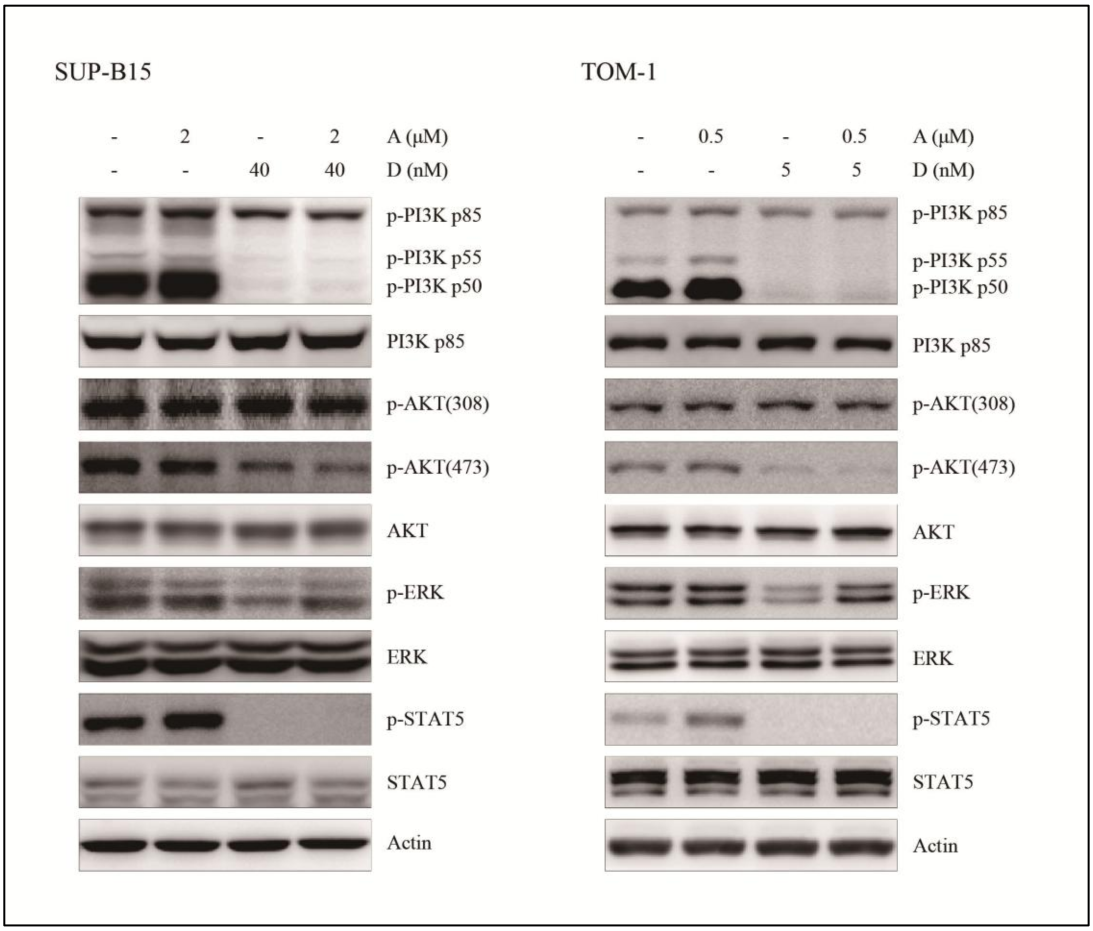

Fig. S3 Effects of ATO and/or Dasatinib on the three main downstream signaling pathways of BCR-ABL1. Total and phosphorylated STAT5, ERK, PI3K and AKT were determined by Western blot after ATO and/or Dasatinib 24 h treatment.

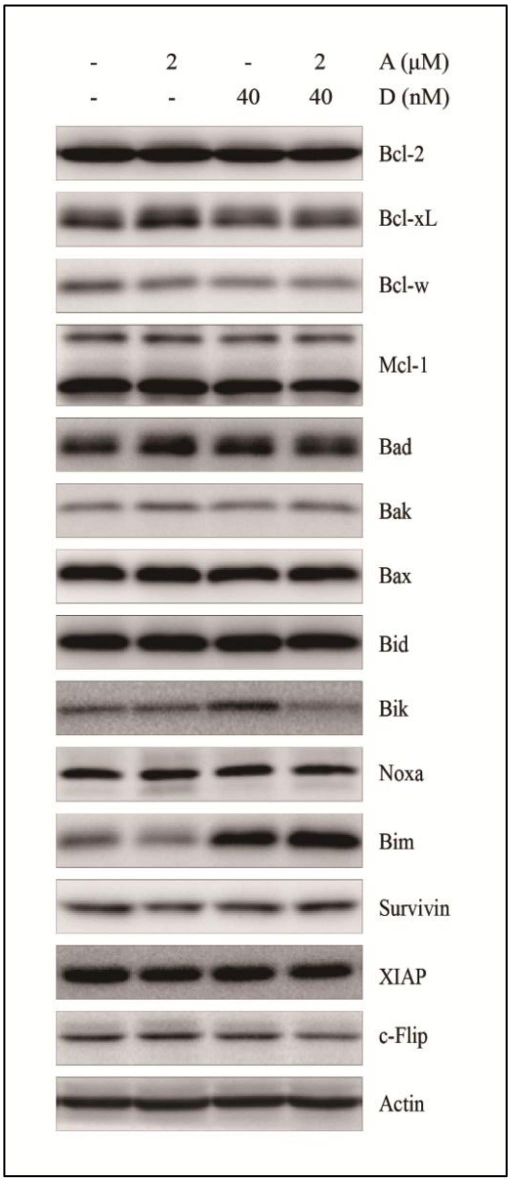

Fig. S4 The expression of proteins of the BCL-2, IAP and Flip families were detected by Western blots after ATO and/or Dasatinib 24 h treatment in SUP-B15 cells.

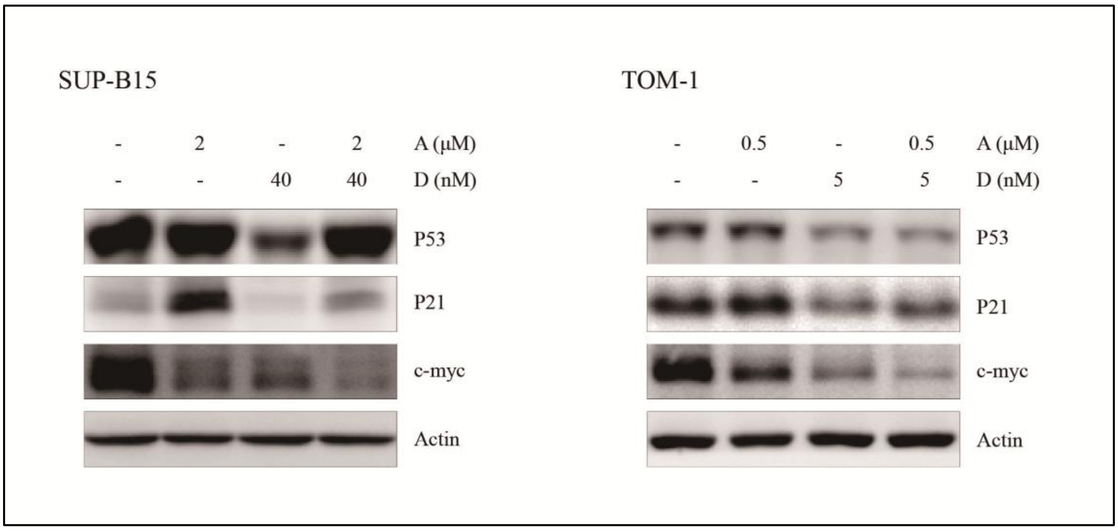

Fig. S5 p53, p21 and c-myc were detected by Western blot after a 24 h treatment with ATO and/or Dasatinib.

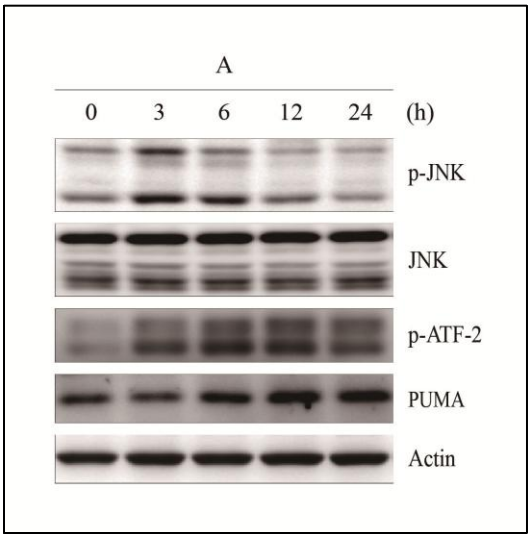

Fig. S6 JNK, p-JNK, p-ATF-2 and PUMA were detected by Western blot after a treatment with ATO (2 μM) for the indicated time courses in SUP-B15 cells.

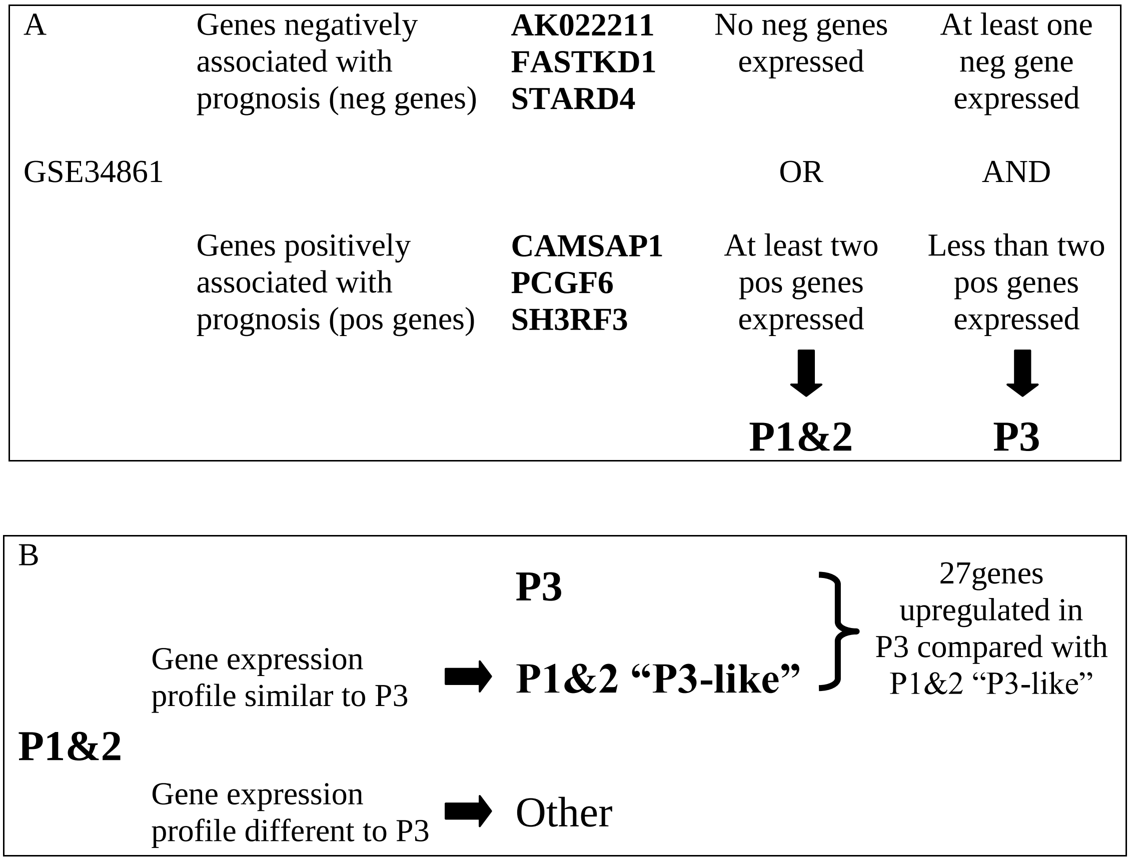

Fig. S7 Strategy for the identification of 27 genes whose activation would be directly related to the aggressiveness of the ALL. **(A)** Tumors in GSE34861 series were stratified into P1&2 and P3 group as a function of the expression of the six genes. **(B)** 27 genes were identified to be up-regulated in P3 tumors compared with P1&2 "P3-like" group.

Table S1 The Combination Index (CI) of ATO and Dasatinib in SUP-B15 and TOM-1 cells

	SUP-B15			TOM-1		
	ATO (μM)	Dasatinib (nM)	CI	ATO (μM)	Dasatinib (nM)	CI
36 h	1.0	20.0	0.8189	0.5	5.0	0.1823
	1.0	40.0	0.8583	0.5	10.0	0.1734
	1.0	60.0	0.8551	0.5	15.0	0.1694
	1.0	80.0	0.8959	0.5	20.0	0.1649
	2.0	20.0	0.6328	1.0	5.0	0.2369
	2.0	40.0	0.5979	1.0	10.0	0.2396
	2.0	60.0	0.5770	1.0	15.0	0.2278
	2.0	80.0	0.5607	1.0	20.0	0.2298
48 h	1.0	20.0	0.4834	0.5	5.0	0.0016
	1.0	40.0	0.5152	0.5	10.0	0.0012
	1.0	60.0	0.4637	0.5	15.0	0.0011
	1.0	80.0	0.4425	0.5	20.0	0.0011
	2.0	20.0	0.3253	1.0	5.0	0.0007
	2.0	40.0	0.2694	1.0	10.0	0.0004
	2.0	60.0	0.1942	1.0	15.0	0.0004
	2.0	80.0	0.1649	1.0	20.0	0.0005

Table S2 Genes up-regulated in P3 vs P1&2 “P3-like”

Gene_Symbol
PARP8
FAM150B
CDK6
ETV6
PTEN
PLD1
MIR155HG
MLF1IP
STARD4
AGPS
CXXC5
ATAD2
LOC100506935
ARPP19
TAPT1
SENP6
SC4MOL
CTGF
DPYD
NFIL3
RGS1
TMED5
ACSL1
IGFBP7
DSTN
FAM91A2
SYTL3
